# 

**Published:** 1835-04-01

**Authors:** 


### MEDICAL QUARTERLY REVIEW.

**No. VII.**

APRIL 1,

1835.

SURGEON GEN'L'S OFFICE
108160
LIBRARY

LONDON:

J. SOUTER, 73, ST. PAUL'S CHURCH-YARD;

SOLD ALSO BY

BURGESS AND HILL; J. CHURCHILL; E. COX; J. HIGHLEY;

RENSHAW AND RUSH, &c.

W. JACKSON, NEW YORK, AGENT FOR THE UNITED STATES.

PRICE FIVE SHILLINGS.

J. AND C. ADLARD, BARTHOLOMEW CLOSE.

